# FerrDb: a manually curated resource for regulators and markers of ferroptosis and ferroptosis-disease associations

**DOI:** 10.1093/database/baaa021

**Published:** 2020-03-27

**Authors:** Nan Zhou, Jinku Bao

**Affiliations:** 1 Affiliated Brain Hospital of Guangzhou Medical University, 36 Mingxin Rd, Guangzhou, 510370, China; 2 Guangzhou Huiai Hospital, 36 Mingxin Rd, Guangzhou, 510370, China; 3 Guangdong Engineering Technology Research Center for Translational Medicine of Mental Disorders, 36 Mingxin Rd, Guangzhou, 510370, China; 4 Key Laboratory of the State Ministry of Education for Bio-Resources and Ecologic Environment, College of Life Sciences, Sichuan University, 29 Wangjiang Rd, Chengdu, 610064, China

## Abstract

Ferroptosis is a mode of regulated cell death that depends on iron. Cells die from the toxic accumulation of lipid reactive oxygen species. Ferroptosis is tightly linked to a variety of human diseases, such as cancers and degenerative diseases. The ferroptotic process is complicated and consists of a wide range of metabolites and biomolecules. Although great progress has been achieved, the mechanism of ferroptosis remains enigmatic. We have currently entered an era of extensive knowledge advancement, and thus, it is important to find ways to organize and utilize data efficiently. We have observed a high-quality knowledge base of ferroptosis research is lacking. In this study, we downloaded 784 ferroptosis articles from the PubMed database. Ferroptosis regulators and markers and associated diseases were extracted from these articles and annotated. In summary, 253 regulators (including 108 drivers, 69 suppressors, 35 inducers and 41 inhibitors), 111 markers and 95 ferroptosis-disease associations were found. We then developed FerrDb, the first manually curated database for regulators and markers of ferroptosis and ferroptosis-disease associations. The database has a user-friendly interface, and it will be updated every 6 months to offer long-term service. FerrDb is expected to help researchers acquire insights into ferroptosis.

**Database URL:** http://www.zhounan.org/ferrdb

## Introduction

Cells are the fundamental building block of multicellular organisms. Cell death is essential for fundamental physiological processes such as development, immunity, and tissue homeostasis (1). Accidental and regulated cell deaths are two subtypes of cell death. Accidental cell death is unavoidable and uncontrollable during which cells die immediately from structural breakdown caused by severe physical, chemical or mechanical stimuli (2). In contrast, regulated cell death can be controlled pharmacologically or genetically by specific intrinsic cellular mechanisms (2). Although the concept of programmed cell death emerged early in the 1960s, the term ferroptosis was coined in 2012 (3). Ferroptosis is an iron-dependent form of regulated cell death. It is morphologically, biochemically and genetically distinct from apoptosis, necroptosis, necrosis, autophagy and other modes of cell death (4, 5). For example, canonical inhibitors against apoptosis do not inhibit ferroptosis induced by the class I ferroptosis inducer erastin or the class II ferroptosis inducer RSL3 (4). Ferroptosis is caused by the accumulation of lipid reactive oxygen species owing to either inactivation of cellular glutathione (GSH)-dependent antioxidant defenses or loss of activity of the lipid repair enzyme glutathione peroxidase 4 (GPX4) (4, 6).

**Table 1:** Annotation data in FerrDb

**Data set**	**Category**	**Annotated from**	**Explanation**
Driver	Regulator	Gene	A driver is a gene that promotes ferroptosis.
Suppressor	Regulator	Gene	A suppressor is a gene that prevents ferroptosis.
Marker	Marker	Gene	A marker is a gene that indicates the occurrence of ferroptosis.
Inducer	Regulator	Small molecule	An inducer is a small molecule that can cause ferroptosis
Inhibitor	Regulator	Small molecule	An inhibitor is a small molecule that can restrict ferroptosis.
Ferroptosis-disease association	Ferroptosis-disease association	Ferroptosis and disease	Ferroptosis has two opposite effects on disease: aggravating an illness while alleviating an unpleasant situation.

After several years of study, ferroptotic cell death was recognized as clinically important. Ferroptosis is being investigated as a therapeutic means of treating human diseases. For example, sorafenib, a first-line drug for hepatocellular carcinoma, depends on ferroptosis to fulfill its cytotoxic effect (7). Ferroptosis’ effect on disease varies with illness. (i) Ferroptosis helps prevent the development of cancer. Ferroptosis is suppressed in hepatocellular carcinoma, blood cancer, colorectal cancer, melanoma, neuroblastoma, head and neck cancer, kidney tumor, glioma, breast cancer, lung cancer, ovarian cancer, pancreatic cancer, rhabdomyosarcoma, cervical carcinoma and prostate cancer, thus facilitating tumor cell proliferation. (ii) Ferroptosis causes injuries to worsen. It has been reported that ferroptosis can exacerbate kidney injury, heart failure, bone marrow injury, brain injury, spinal cord injury and intestinal ischemia/reperfusion injury. (iii) Ferroptosis is able to aggravate degenerative diseases. There is evidence that ferroptosis can result in Huntington’s disease, rapid motor neuron degeneration, paralysis, Parkinson’s disease, stroke and Alzheimer’s disease. (iv) Ferroptosis contributes to infectious diseases. Acute lymphocytic choriomeningitis virus and *Leishmania* major parasite infections benefit from ferroptosis (8). (v) Friedreich’s ataxia, hemochromatosis, asthma, cardiomyopathy, temporal lobe epilepsy, alcoholic steatohepatitis and alcoholic liver are worsened by ferroptosis. (vi) Ferroptosis appears to exert different impacts on fibrosis-associated diseases; for example, ferroptosis is favorable for radiation-induced lung fibrosis but unfavorable for liver fibrosis (9, 10).

Given ferroptosis’ critical role in mammalian development, homeostasis and disease, the number of publications in this field continues to increase, from a few publications in 2012 to hundreds of publications per year. These published articles contain essential information about how ferroptosis is regulated by genes and small molecules and the effects of ferroptosis on disease. However, collecting such information is time-consuming and laborious because substantial literature review is required. A high-quality knowledge base is fundamental for biological research. In this study, we collected genes and small molecules and then annotated them as regulators and markers of ferroptosis. We also evaluated ferroptosis-associated diseases and subsequently annotated ferroptosis’ effect on diseases. Finally, we built FerrDb, the first database that aggregates ferroptosis regulators and markers and ferroptosis-disease associations.

## Methods and materials

### Article collection

To obtain literature on ferroptosis, we searched the PubMed database (https://www.ncbi.nlm.nih.gov/pubmed) using the term ‘ferroptosis’ on 12 July 2019. When our manuscript was under review, we also searched the PubMed database on 20 February 2020 to find all ferroptosis articles of year 2019. All ferroptosis-related articles found in PubMed were downloaded. We then read these articles to identify genes, small molecules and diseases related to ferroptosis. Afterwards, we performed annotation for regulators, markers and effects of ferroptosis on disease according to experimental evidence from the collected publications.

### Annotation strategy

Annotation data sets in FerrDb belong to three categories (Table 1). Genes were annotated as drivers, suppressors and markers. Small molecules were annotated as inducers and inhibitors. Drivers, suppressors, inducers and inhibitors are regulators of ferroptosis: drivers and inducers positively regulate ferroptosis, while suppressors and inhibitors negatively regulate ferroptosis. Markers do not regulate ferroptosis, but they indicate the occurrence of ferroptosis. Ferroptosis affects the development of disease in two ways. Ferroptosis was then annotated to either aggravate or alleviate an illness.

**Table 2:** Confidence levels for gene annotation

**Confidence**	**Explanation**
Validated	The most reliable: requires convincing evidence from strict tests such as pharmacological or genetic inhibition or activation tests.
Screened	No strict validation: only high-throughput data is available.
Predicted	No strict validation: in the source article, the original authors drew their conclusions based on computational results or their own knowledge.
Deduced	No strict validation: we, the curators, inferred the gene’s function based on our understanding of the original article.

To be annotated as a ferroptosis regulator, genes and small molecules must possess explicit evidence to prove their regulatory role in ferroptosis. This kind of evidence is generally represented by an author statement of the role of the regulator in an original article. Genes that only undergo abundance, modification or stability change or are merely a component of a functional signaling axis or interaction network were annotated as markers. To annotate ferroptosis’ effect on diseases, evidence based on a growth test in cell lines or animal models was required.

In comparison with revealing a small molecule’s role, confirming a gene’s function is more challenging. We therefore dedicated more effort to gene annotation. A confidence level was assigned to each annotation to indicate its reliability (Table 2). Experimental reproducibility is correlated with results consistency, so the number of experiments was used as a score of the accuracy of the regulatory role of annotated genes. Critical cases (e.g. article retraction, conflicting results) that may affect the annotation reliability were highlighted by a caution statement. Other noteworthy information (e.g. inconsistent gene symbols) that seems less likely to impair annotation quality was denoted with a remark.

**Figure 1:**
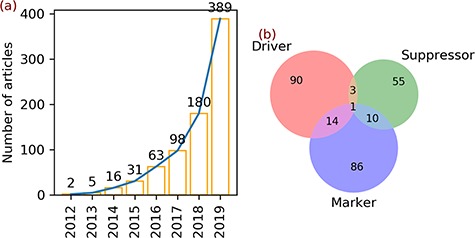
Articles and gene annotation. (**a**) The increasing trend in the number of ferroptosis articles in PubMed. (**b**) Intersection of three annotation data sets of genes.

**Table 3:** Statistics of data in FerrDb

**Data set**	**Count**	**# annotations**
*Gene*	259^a^	/
• Driver	108^a^	150
• Suppressor	69^a^	109
• Marker	111^a^	123
*Small molecule*	76	/
• Inducer	35	54
• Inhibitor	41	46
*Ferroptosis-disease association*	95	135
• Ferroptosis aggravates disease	49	58
• Ferroptosis alleviates disease	46	77

^a^Drivers (108) + suppressors (69) + markers (111) = 288, which is larger than the gene count (259) because of 28 multi-annotated genes, as shown in Figure 1b.

**Figure 2:**
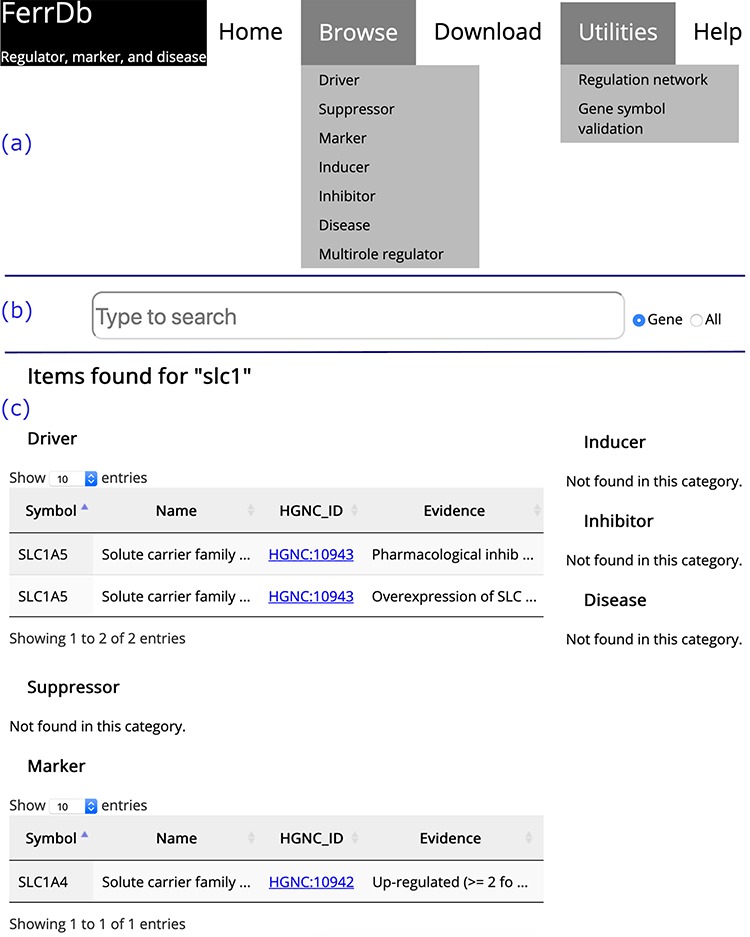
Overview of the FerrDb interface. (**a**) Navigation bar. (**b**) Search box. (**c**) Results of an example search. Tables in (c) are truncated.

**Figure 3:**
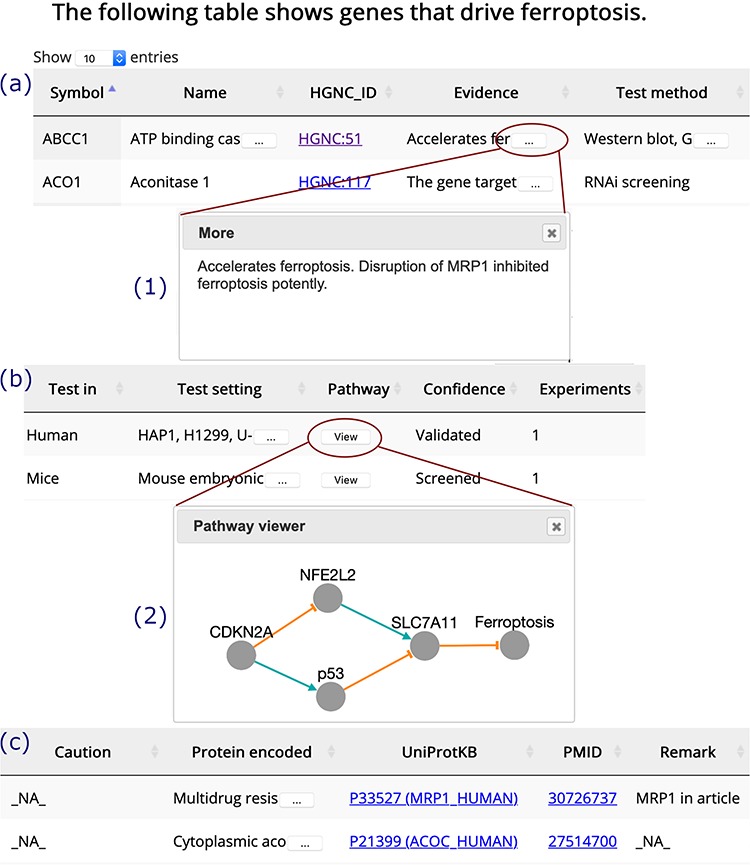
A browser page of drivers. Table columns are split into three parts, (**a**), (**b**) and (**c**). Inset (1) shows the whole content of truncated text. Inset (2) visualizes the regulation pathway of an example gene. The orange T-shaped edge denotes suppression, and the green arrow denotes promotion.

**Figure 4:**
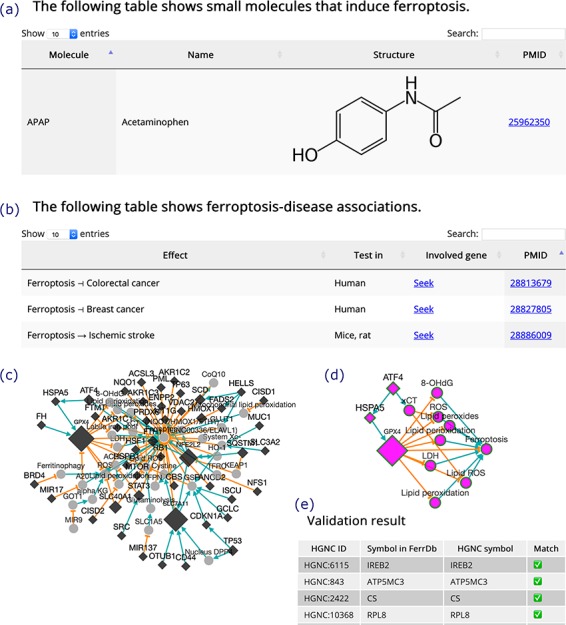
(**a**) An inducer. (**b**) Ferroptosis-disease associations. The T-shaped line denotes alleviation, while the arrow denotes aggravation. (**c**) Regulation network. Diamonds denote regulators of ferroptosis. Circles denote unclassified regulatory elements. Orange T-shaped lines denote inhibition. Green arrows denote promotion. Node size represents the number of experiments, where larger nodes reflect a greater number of experiments. (**d**) Sub-network of ATF4, shown as an example. (**e**) A truncated result table of gene symbol validation. The white check mark in green box indicates success of validation. The red cross indicates failure of validation.

### Development of FerrDb

FerrDb was developed with HTML, CSS and JavaScript. JavaScript libraries jQuery (https://jquery.com/) and jQuery UI (https://jqueryui.com/) were used to enhance interactive features of the website. DataTables (https://datatables.net/) was used to display the data in tables. Cytoscape.js was used to visualize regulation networks on the web page (11). Annotation data sets were stored in JSON files, which were used as the core database to drive FerrDb. To keep gene symbols in FerrDb consistent with those in the HGNC database (https://www.genenames.org/), HGNC’s REST API (application programming interface) was used to verify genes in FerrDb. The FerrDb website was hosted by an Apache (http://www.apache.org/) web server that was deployed as an Amazon AWS EC2 instance (https://aws.amazon.com/) running Ubuntu 16.04 LTS (https://ubuntu.com/).

## Results

### Trends in ferroptosis research

A total of 784 articles regarding ferroptosis were found upon searching the PubMed database. In 2012, the term ferroptosis was defined and used for the first time to describe a new form of regulated cell death. In the same year, two articles were published (Figure 1a). In the next 3 years, the number of ferroptosis articles increased slightly to 5 in 2013, 16 in 2014 and 31 in 2015 (Figure 1a). Afterwards, the number of articles continued to increase and reached 389 in 2019 (Figure 1a).

### Statistics of data in FerrDb

Two hundred fifty-nine genes were found in the 784 ferroptosis articles (Table 3). Annotation of these genes revealed 108 drivers, 69 suppressors and 111 markers (Table 3). Some genes belong to multiple annotation sets, with 14 genes in both drivers and markers, 3 genes in both drivers and suppressors, 10 genes in both suppressors and markers and 1 gene in all sets (Figure 1b).

Of the 784 articles that were evaluated for this study, 76 small molecules were found to play a role in the regulation of ferroptosis. As can be seen from Table 3, 35 of these small molecules were annotated as inducers, and the others were annotated as inhibitors.

Table 3 also displays 95 ferroptosis-disease associations discovered from the collected articles. Forty-nine of the associations indicate that ferroptosis plays a role in exacerbating a disease and the other associations indicate that ferroptosis exerts effects in alleviating the severity of an illness.

### Introduction to FerrDb

On the home page, links to other FerrDb pages are provided in the navigation bar (Figure 2a). There is also a search box on the home page (Figure 2b). The user can determine the text in which to search by selecting either the ‘Gene’ or ‘All’ radio button. The default is set to search by ‘Gene’. The search results are shown on a new page. Found items are displayed in a table under the corresponding data set; otherwise, ‘Not found’ is displayed as a note (Figure 2c).

The ‘Browse’ drop-down menu in the navigation bar provides connections to annotation data sets. Drivers, suppressors and markers are shown in tables in a similar format, and the browser page of drivers is shown as an example (Figure 3). Genes are shown in the rows, with annotations shown in the columns. Clicking on the name of a column will sort the rows, and the selected column will be highlighted. The text is truncated if the cell is not wide enough to display all of the text with an ellipsis button displayed at the end (inset (1) in Figure 3). To view a gene’s regulation pathway in ferroptosis, a ‘View’ button in the ‘Pathway’ column is provided, as illustrated in the inset (2) in Figure 3.

Inducers and inhibitors are shown in tables in the same format, and the browser page of inducers is shown in Figure 4a as an example. In the browser page that shows ferroptosis-disease associations (Figure 4b), the ‘Effect’ column shows whether ferroptosis exacerbates or alleviates a disease. The search box at the top right of the table can be used to search the content in the table. A ‘Seek’ link in the ‘Involved gene’ column can be used to identify ferroptosis genes involved in a disease.

In addition to the regulation pathway of respective genes, FerrDb can integrate individual pathways to generate a regulation network (Figure 4c). This feature can be accessed via the ‘Regulation network’ tool on the ‘Utilities’ menu in the navigation bar. The network can be built from regulation pathways of drivers and/or suppressors. The network is interactive, and the user can view the sub-network of a specific gene by clicking on its node (Figure 4d). The ‘Gene symbol validation’ tool in the ‘Utilities’ menu in the navigation bar provides an easy way to validate gene symbols in FerrDb against the official HGNC symbols (Figure 4e). Any inconsistencies that appear will be identified and fixed within 2 weeks.

### Application example: finding a gene valuable to future study

**Figure 5:**
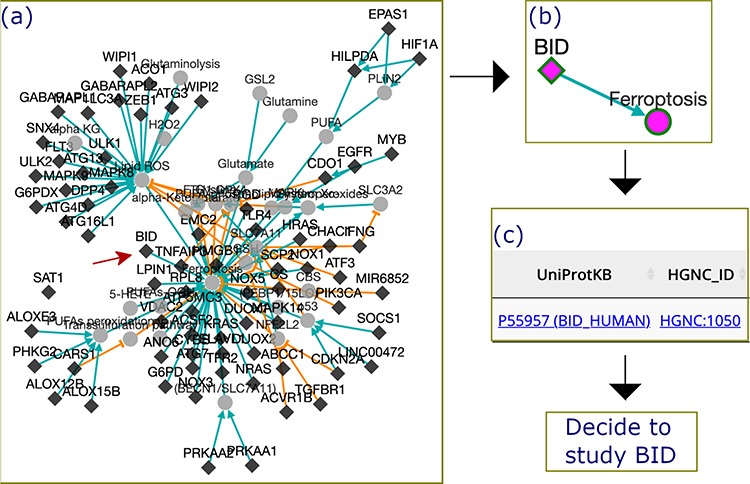
The process by which FerrDb was used to identify BID as a valuable gene for future study. (**a**) A regulation network integrating drivers’ pathways. Drivers with more than one experiment were removed from the network. (**b**) BID pathway in ferroptosis. (**c**) Links to BID resources.

In this example, we used FerrDb to discover BID, a ferroptosis driver that is worth further research. First, we filtered out genes with more than one experiment from the drivers’ regulation network, and genes that had only been studied once were retained (Figure 5a). Second, we scrutinized the filtered network and found that the BID node directly linked with the ferroptosis node (Figure 5b). This means that BID apparently promotes ferroptosis, although the interacting molecules remain obscure. Third, we used the HGNC and UniProt links provided by FerrDb to read BID annotation from other resources (Figure 5c). With these steps, we understood that the BID gene encodes the BID protein which belongs to the pro-apoptotic BCL-2 family (12). Hence, we hypothesize that ferroptosis can interplay with apoptosis via a BID-mediated signaling network, but we are currently lacking evidence to validate this hypothesis. Therefore, it is worthwhile to study the molecular mechanisms of BID in regulating ferroptosis in the future.

## Discussion

The mechanism of ferroptosis is highly complex. In oncology studies, some genes have been identified that promote one type of cancer but suppress another type (13). Similarly, we found that this phenomenon also exists in ferroptosis. For example, Jiang and colleagues discovered that p53 can induce ferroptosis upon stresses induced by reactive oxygen species (14), while Xie *et al*. found that TP53 limits erastin-induced ferroptosis by blocking dipeptidyl-peptidase-4 (DPP4) activity in a transcription-independent manner (15). Therefore, understanding the regulatory role of genes based on biological context is necessary to illustrate the complex mechanisms of ferroptosis.

It should be noted that our search strategy may exclude some articles that should be included. For instance, articles indexed in other databases or articles lacking the word ‘ferroptosis’ but that are mechanistically relevant to ferroptosis would not be reported. Considering that ferroptosis was not well defined until 2012, the number of articles that use other words to describe ferroptosis should be small (4). Because PubMed automatically matches search terms against a subject translation table (including for example, MeSH (Medical Subject Headings)), most target articles would be found. Many articles in other databases may be ignored by the PubMed search, and we plan to address this limitation in future updates of FerrDb by aggregating articles from multiple sources.

In the study, annotations were generated based on the assumption that the collected literature report correct results. We acknowledge that this assumption may introduce some errors because unreliable scientific claims might exist in original studies, especially in life sciences research which is beset by low reproducibility rates (16). Our annotation strategy does not prevent potential errors in original studies, but error-prone situations were emphasized by caution and remark tags. To demonstrate the reliability of the annotations, confidence levels were assigned each annotation. Moreover, the number of experiments performed for each gene was also used as an indicator to reflect the accuracy of the gene’s function. These strategies help ensure the high reliability and quality of data in FerrDb.

Researchers who are interested in ferroptosis can use the annotation data to, for example, (i) design new ferroptosis inducers or inhibitors, (ii) study how ferroptosis acts in a given disease, (iii) study the mechanisms of cancer cell resistance to anti-cancer drugs such as sorafenib, (iv) treat degenerative diseases by blocking ferroptosis and (v) apply enrichment analysis on a set of genes to determine if those genes are enriched in the ferroptotic process.

## Conclusion

We have developed FerrDb, the first database of experimentally validated ferroptosis regulators and markers and ferroptosis-disease associations. Annotations were generated from currently available ferroptosis articles in PubMed. In order to provide high-quality and long-term service, FerrDb will be updated every 6 months. This first release of FerrDb can only be used to browse and fetch data. In future releases, we plan to extend FerrDb to integrate online analysis tools to provide a powerful platform for biologists to study the complexities of ferroptosis.

## Data and code availability

The published article includes all data and code generated or analyzed during this study.

## Author contributions

J.B. conceived and supervised the study. N.Z. carried out this project and wrote the draft of the manuscript. J.B. revised and reviewed the manuscript.
